# Design, Fabrication, and Performance Evaluation of Portable and Large-Area Blackbody System

**DOI:** 10.3390/s20205836

**Published:** 2020-10-15

**Authors:** Ji Yong Bae, Won Choi, Suk-Ju Hong, Sangyeon Kim, Eungchan Kim, Chang-Hyup Lee, Yun-hyeok Han, Hwan Hur, Kye-Sung Lee, Ki Soo Chang, Geon-Hee Kim, Ghiseok Kim

**Affiliations:** 1 Division of Scientific Instrumentation and Management, Korea Basic Science Institute, 169-148 Gwahak-ro, Yuseong-gu, Daejeon 34133, Korea; baejy@kbsi.re.kr (J.Y.B.); hurhwan@kbsi.re.kr (H.H.); kslee24@kbsi.re.kr (K.-S.L.); ksc@kbsi.re.kr (K.S.C.); kgh@kbsi.re.kr (G.-H.K.); 2 Department of Rural Systems Engineering, Seoul National University, 1 Gwanak-ro, Gwanak-gu, Seoul 08826, Korea; fembem@snu.ac.kr; 3 Global Smart Farm Convergence Major, Seoul National University, 1 Gwanak-ro, Gwanak-gu, Seoul 08826, Korea; oxycle@snu.ac.kr (E.K.); dlckdguq731@snu.ac.kr (C.-H.L.); 4 Department of Biosystems Engineering, Seoul National University, 1 Gwanak-ro, Gwanak-gu, Seoul 08826, Korea; hsj5596@snu.ac.kr (S.-J.H.); yskra@snu.ac.kr (S.K.); 5 ISAE-Supaéro, Université de Toulouse, 10 avenue Edouard Belin, 31055 Toulouse, France; yunhyeok.han@gmail.com; 6 Research Institute of Agriculture and Life Sciences, Seoul National University, 1 Gwanak-ro, Gwanak-gu, Seoul 08826, Korea

**Keywords:** portable and large-area blackbody system, infrared sensor, finite elements analysis, signal transfer function, noise equivalent temperature difference

## Abstract

In this study, a portable and large-area blackbody system was developed following a series of processes including design, computational analysis, fabrication, and experimental analysis and evaluation. The blackbody system was designed to be lightweight (5 kg), and its temperature could exceed the ambient temperature by up to 15 °C under operation. A carbon-fiber-based heat source was used to achieve a uniform temperature distribution. A heat shield fabricated from an insulation material was embedded at the opposite side of the heating element to minimize heat loss. A prototype of the blackbody system was fabricated based on the design and transient coupled electro-thermal simulation results. The operation performance of this system, such as the thermal response, signal transfer function, and noise equivalent temperature difference, was evaluated by employing an infrared imaging system. In addition, emissivity was measured during operation. The results of this study show that the developed portable and large-area blackbody system can be expected to serve as a reliable reference source for the calibration of aerial infrared images for the application of aerial infrared techniques to remote sensing.

## 1. Introduction

In recent years, the demand for cutting-edge technology related to the fourth industrial revolution has been increasing. As a result, extensive research has been performed in this area, including research on the use of drones in numerous fields. The earliest drones were mostly developed for military purposes. However, since the 2000s, they have been widely used in various civilian fields such as surveying, logistics, photography, agriculture, environment, and civil engineering. In particular, there has been a rapid increase in research related to unmanned aerial vehicle (UAV) technologies in the private sector, e.g., surveying, broadcasting, photography, crop growth and development assessment, forest and ecosystem diagnosis, and facility safety inspection [1,2,3,4,5].

Previously, in the case of aerial images captured by UAVs, most image sensors (cameras) only captured images within the visible spectrum. However, with the progress of image sensors and UAV technologies, image sensors that can measure signals in multiple bands are being developed. In addition, thermal imagers for UAVs are being developed. For these reasons, research on remote sensing and inspection using UAVs with infrared thermal imaging technology has been increasing rapidly [6,7,8,9]. Berni et al. [10] performed vegetation monitoring using a UAV to acquire thermal images and multispectral images in the 400–800 nm bands. They verified the effectiveness of the UAV system for vegetation monitoring by calculating vegetation indices, chlorophyll content, and the photochemical reflectance index using the acquired images and comparing them with actual values. Bellvert et al. [11] proposed a method for measuring the crop water stress index (CWSI) using a UAV. The CWSI was calculated using the aerial thermal images captured by the UAV, based on the ground and the leaf water potential value. Then, the calculated CWSI was utilized to confirm the ideal time and altitude for image acquisition. The threshold CWSI was set using the calculated CWSI map, and irrigation management on each interesting zone could be performed.

Early infrared thermal imaging technology was used for military purposes in the early 1900s and in the private sector from the 1960s. Since then, it has been applied to various fields and has progressed rapidly with the development of sensor technology. Currently, infrared thermal imaging technology is being investigated and developed in numerous fields in the form of infrared thermography. Infrared thermal imaging technology not only measures the surface temperature of an object but also provides information about heat intrusions and heterogeneity in the interior or subsurface of the object [12,13,14]. This information can be used to to inspect the condition and uniformity of the object. Therefore, in the case of living biological resources or agricultural products, it is possible to diagnose diseases and analyze physical and chemical integrity and the presence of damage. Furthermore, extensive research on non-destructive remote testing has been conducted in the fields of aerospace, automotive industries, construction, and civil engineering [15,16,17,18,19].

Infrared thermal imaging technology provides several advantages such as non-destructive, non-contact, and full-field imaging, and rapid inspection capabilities. However, this technology has a few inherent limitations. For instance, the temperature distribution in an object varies depending on the intrinsic characteristics of the object (color, material, emissivity, etc.); measured temperature may be affected by the environment; and the spatial resolution of thermal imaging is lower than that of images captured within the visible spectrum. Various infrared thermal image processing technologies have been developed to overcome these limitations, and thermal image correction techniques and devices have been developed to minimize the influence of the environment. In addition, the increase in the use of infrared cameras in UAVs for civilian applications, existing infrared thermal imaging technology has also been applied to aerial infrared thermal imaging. However, it is more important to consider the measurement distance or altitude and environment in aerial infrared thermal imaging technology compared to conventional infrared thermal imaging technology. Therefore, it is necessary to calibrate infrared image signals according to the environment and measured altitude for remote inspection using aerial infrared thermal imaging.

Blackbody systems are generally used to calibrate infrared radiation measurement devices, such as infrared thermal imaging sensors or infrared cameras, or to calibrate thermal image information. A blackbody system is defined as a precise radiant heat generator with an emissivity close to 1, and it is used to calibrate broadband infrared radiant heat sensors [20]. Emissivity is a key parameter that represents the ability of an object to emit infrared energy. Commercial blackbody systems typically use liquids, such as oil or water, as refrigerants. As these systems contain an internal vacuum chamber, they are commonly used in laboratory environments where temperature and humidity are controlled [21,22,23,24]. Lee et al. [21] developed a blackbody system that operates under vacuum conditions (2.67 × 10^−2^ Pa) to reduce its temperature uncertainty, which can be caused by vapor condensation at low temperatures below 273.15 K, and thermophysically evaluated the blackbody system using a simplified 3D model including a radiator, heat sink, heat shield, and cold shield by performing finite elements analysis using the extended Stefan–Boltzmann’s rule, and the infrared radiating performance of the developed system was analyzed using an infrared camera system. Chu et al. [22] developed a cylindrical blackbody system with a temperature stability of ±0.3 °C using a new ammonia heat pipe reference source. This blackbody system operates in a temperature range of −40–50 °C with an emissivity of 0.9993. Palchetti et al. [23] developed a blackbody system that can be easily used in a laboratory. The blackbody system operates over a broad spectral range of 100–1400 cm^−1^ and in a temperature range of −10–120 °C, with an emissivity above 0.9999 and a temperature error of 0.5 K. Morozova et al. [24] developed a cavity-type cold blackbody system using a vacuum variable-low-temperature blackbody (VTBB) and BB100K1. The system operates at temperatures ranging from −60–90 °C, and it has an emissivity of 0.9997 when the VTBB is used and 0.997 when BB100K1 is used. The temperature errors are ±40 mK and ±50 mK for the VTBB and BB100K1, respectively. Existing commercially developed blackbody systems are used in indoor environments, where temperature and humidity can be controlled easily, and the measurement distance between a blackbody system and a thermal image sensor is within a few meters. However, it is almost impossible to apply existing commercial blackbody systems to obtain aerial infrared thermal images outdoors because the measurement altitude exceeds tens of meters and constant temperature and humidity cannot be maintained. Therefore, a new blackbody system is required to analyze the measurement accuracy of the infrared thermal image sensors mounted on UAVs and to calibrate the thermal images of objects obtained outdoors. Such a blackbody system must be portable so that it may be used at different sites. Additionally, it must be a relatively large area so that it may be possible to acquire thermal images at altitudes of several tens of meters. However, extremely few researchers have investigated this type of portable and large-area blackbody system required for aerial infrared thermal imaging technology. Moreover, there has been negligible research on the development and evaluation of such portable and large-area blackbody systems worldwide [25,26,27,28].

Fowler, J.B [25] developed a water bath-based blackbody source which has a wide (10.8 cm) diameter aperture, an extended conical cavity section. The water temperature stability of the blackbody source is ±2 mK. The temperature uniformity of the water volume is ±2.0 mK at the lowest temperature in its operating range and ±5.0 mK at the high end of its operating range of 278 to 353, as measured using the resistance thermometry. Park et al. [27] constructed a large aperture blackbody for calibrating radiation thermometers and infrared radiometers with a wide field of view in the temperature range between 10 °C and 90 °C. The blackbody is a 1 m long cylindro-conical cavity with a diameter of 1.1 m. Its conical bottom has an apex angle of 120°. To achieve good temperature stability and uniformity, the cavity is integrated to a water-bath to which the pressurized water is supplied from a reservoir. To reduce the convection heat loss from the cavity to the ambient, the cavity is purged of the dried air that passes through a coiled tube immersed in the reservoir. For an uncertainty evaluation of the large aperture blackbody, its temperature stability was measured by using a reference radiation thermometer and a platinum resistance thermometer, and its radiance temperature distributions on the aperture plane were measured by using a thermal camera. Measuring the spectral emissivity of the coating material, the effective emissivity of the blackbody was calculated to be 0.9955 from 1 μm to 15 μm. Miklavec et al. [28] constructed a large aperture blackbody, covering the complete FOV of the thermal imager and having better stability and non-uniformity than the thermal sensitivity of the imager. The blackbody calibration bath was designed on a hypothesis analogous to the multi-zone furnace, where the role of electrical heaters was superseded by electrically controlled valves. The experimental work showed that the designed system enables traceable calibration of thermal imagers in the temperature range from 10 to 70 °C with the expanded uncertainty of 0.2 °C.

In this study, we designed a portable and large-area blackbody system that can be applied to various fields. The heat radiation area of the system is larger than that of conventional blackbody systems. The effectiveness of the design for the portable and large-area blackbody system was confirmed by analyzing thermal responses, such as the uniformity of the temperature distribution, radiant heat range, and steady-state arrival time, through transient coupled electro-thermal simulations. Thereafter, a prototype of this blackbody system was fabricated. The operation performance of the prototype system was verified by evaluating transient thermal responses, comparing them with simulation results, and analyzing and the signal transfer function (SiTF) and noise-equivalent temperature difference (NETD) using an infrared thermal image sensor. Moreover, the emissivity of the system was evaluated on the black surface of the heat radiator at densely distributed measurement points.

## 2. Design

**Figure 1:**
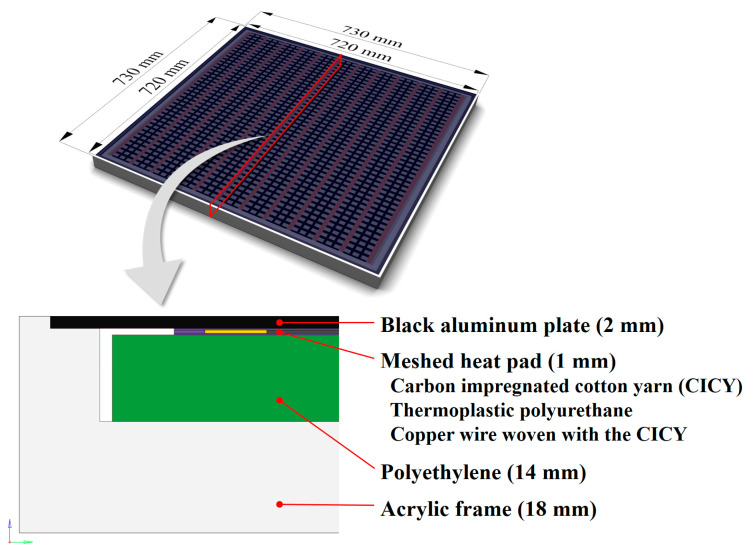
Schematics of the portable and large-area blackbody system: upper part shows a three dimensional perspective view of the blackbody system, and bottom part represents a cross-sectional view with a detailed description of structure.

Figure 1 shows the schematic of the portable and large-area blackbody system designed in this study. The system is composed of (1) a radiator fabricated from an aluminum plate, (2) a carbon-fiber-based meshed heat pad, (3) an insulating material fabricated from polyethylene foam, and (4) an outer frame of an acrylic material. The width, length, and height of the blackbody system are 730, 730 and 50 mm, respectively, and its weight is 5 kg, which makes it portable. Moreover, a variable direct-current constant-voltage supply device (constant-voltage controller) is employed to generate and maintain the target radiant heat. The heat radiator of the blackbody system is fabricated from aluminum. The width, length, and thickness of the radiator are 720, 720, and 2 mm, respectively, and its coefficient of thermal conductivity is 240 ± 5 W/m⋅K. The surface of the radiator is anodized and coated with black antireflective paint to improve its durability and corrosion resistance and increase emissivity. The operating range of the radiant heat of the blackbody system is designed to increase the temperature of system by up to 15 °C compared to the ambient temperature, which is 25 °C. A heat source fabricated from carbon-fiber-based cotton mesh and copper wire is employed in the blackbody system to generate radiant heat on the surface of the system. This heat source (thickness: 1 mm) consists of cotton yarns that are woven into a grid pattern and impregnated with a carbon liquid solution. Furthermore, the copper wires are inserted into the cotton yarns with regular intervals and coated with insulation created using a polyurethane material. Heat is generated by the ohmic heating effect, which occurs at the copper wires and spreads through the carbon-impregnated fibers between the copper wires. The grid pattern of a heat source allows for a uniform heat distribution and a relatively high heating speed over a large area.

As shown in Figure 1, the meshed heat pad and aluminum plate are integrally bonded together to minimize the loss of radiant heat conducted from the heating element to the radiator by preventing the condensation of water vapor due to the porous layer present in the contact area. In addition, the opposite side of the heating element is integrally bonded with a heat shield fabricated from an insulation material to minimize heat loss in the direction opposite to the heating element. The insulation material applied to the blackbody system is a molded foam material created using a polyethylene resin, and the thermal conductivity of the molded foam material is 0.3 ± 0.1 W/m⋅K. Moreover, the opposite surface of the insulation material is coated with an aluminum film to minimize the radiant heat loss caused by the influence of the external atmosphere. The resolution of the temperature controller used in the blackbody system is 10 mK. Four thermistor-type temperature sensors are installed symmetrically at 90° intervals to monitor the surface radiant heat of the blackbody system in real time.

## 3. Numerical Simulations

The suitability of the proposed design was confirmed by performing coupled electro-thermal simulations to analyze the thermal responses of the blackbody system heated by electric current (referred to as Joule heating), based on the methods of Bae et al. [29]. Based on the geometric dimensions of the design (see Figure 1), a 3D finite element (FE) model of the entire blackbody system was constructed, including the black aluminum plate, meshed heat pad, insulating material, and acrylic frame. The meshed heat pad was geometrically complex because it consisted of a carbon-impregnated cotton yarn (CICY), thermoplastic polyurethane, and a copper wire woven with the CICY. Hence, its structure was simplified to reduce computational cost (see Figure 1). Fine FE meshes composed of 672,562 nodes and 612,156 hexahedral elements were used for the entire blackbody system. Therefore, it was considered that the total number of elements in the constructed FE model was sufficient for reliably solving this problem without further refinement or increase in the number of elements.

The electro-thermal behavior of the blackbody system was simultaneously governed by the heat equation and Maxwell equation. The temperature distribution within the blackbody system was calculated using the transient heat equation, which is expressed as:

(1) $$\rho C_{p}\frac{\partial T}{\partial t}=\nabla \cdot [k\nabla T]+Q_{J},$$

where *ρ* (kg/m^3^), *C_p_* (J/kg·K)*,* and *k* (W/m·K) are the density, specific heat capacity at constant pressure, and thermal conductivity, respectively. *T* = *T*(*x*,*y*,*z*,*t*) (K) is the temperature in Cartesian coordinates (*x*,*y*,*z*), *t* is time, and *Q_J_* (W/m^3^) is the volumetric heat source generated by Joule heating. The Joule heat, *Q_J_*, generated by the ohmic losses that occur owing to the resistance to the flow of electric current in conductors is defined by:

(2) $$Q_{J}=\frac{dP_{loss}}{d\nu_{vol}}=JΕ,$$

where *P_loss_* (W) is the power lost from the electric field and *ν_vol_* (m^3^) is volume. **J** (A/m^2^) and **E** (V/m) are the current density and electric field, respectively. Based on Ohm’s law and Maxwell’s equations, the ohmic losses, **JE**, can be rewritten as:

(3) $$JΕ=\sigma {\left|Ε\right|}^{2}=\sigma {\left|\nabla V\right|}^{2},$$

where *σ* (S/m) is the electrical conductivity and *V* (volt) is the electric potential.

In the FE analysis, suitable electric and thermal boundary conditions were applied to imitate the Joule heating process of the blackbody system. The initial conditions for the electric potential and temperature of the entire system are:

(4) $$\begin{array}{l} V|{}_{t=0}=V_{0}=0\;V\\ T|{}_{t=0}=T_{0}=298.15\;K \end{array}$$

For all outer surfaces except the bottom surface of the acrylic frame, a Robin boundary condition was applied as the convective condition:

(5) $$-k\frac{\partial T(r,t)}{\partial n}=h(T_{s}-T_{\infty }),$$

where *h* is the convective heat transfer coefficient (W/m^2^·K), and *T_s_* and *T_∞_* represent the temperature at the body surface and the ambient temperature, respectively. Assuming natural convection conditions at room temperature, *h* and *T_∞_* were considered to be 3 W/m^2^·K and 298.15 K, respectively. In addition, the following radiative condition was applied to the upper surface of the black aluminum plate:

(6) $$-k\frac{\partial T(r,t)}{\partial n}=\varepsilon \sigma_{sb}(T_{s}^{4}-T_{\infty }^{4}),$$

where *ε* is the emissivity of the surface and *σ_sb_* is the Stefan–Boltzmann constant. Assuming an almost perfect blackbody system, emissivity was set as 0.985. The electric insulation condition (**n**·**J** = 0, where **n** is the boundary normal vector) was applied to all outer surfaces of the calculation domain for the CICY and the copper wire woven with the CICY. A constant bias voltage (ranging from 10–22.5 V) was applied to the ends of the copper wire, while the ground condition (*V* = 0) was applied to the opposite ends of the copper wire.

All components of the blackbody system were assumed to be continuous, homogeneous, and isotropic. Temperature-dependent thermal properties were applied to the black aluminum plate and thermoplastic polyurethane. The electrical and thermal properties of the CICY and the copper wire woven with the CICY were provided by the manufacturer of the meshed heat pad (Orientaldream, Inc., Hwasung, Korea). All material properties used for the FE analysis are listed in Table 1. Transient coupled electro-thermal simulations were run for 3600 s using the commercial FE software COMSOL Multiphysics (Version 5.4, COMSOL Inc., Palo Alto, CA, USA).

## 4. Experimental Performance Evaluation of Portable and Large-Area Blackbody System

**Figure 2:**
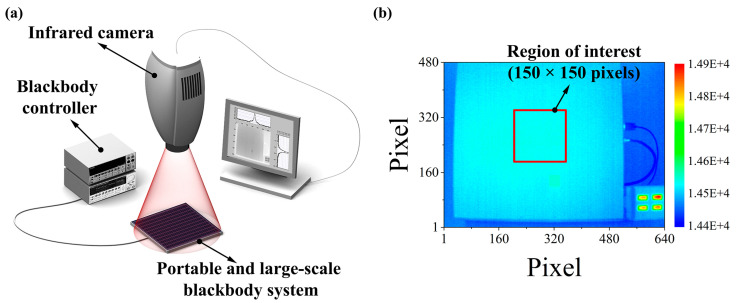
(**a**) Schematic of the experimental configuration for infrared thermal imaging; (**b**) thermal image with the region of interest, in which 100 frames were measured for calculating the noise equivalent temperature difference (NETD) of the portable and large-area blackbody system.

To verify the operation performance of the prototype of the portable and large-area blackbody system, we evaluated the transient thermal responses and analyzed two factors: the SiTF and NETD, using an infrared thermal imaging system (see Figure 2a). The SiTF is generally used for providing information such as the linearity, dynamic range, saturation, and gain of a detection sensor and for calculating the NETD. The NETD refers to the temperature deviation that can measure the smallest thermal image signal, or the smallest temperature value that can be measured by an infrared camera. The infrared camera (ImageIR® 8300, InfraTec GmbH, Dresden, Germany) used in the experimental setup played a key role in the performance evaluation, and it had the following specifications: a resolution of 640 × 480 pixels, a spectral range sensitivity of 1.8–5.5 μm, and a thermal measurement range of −40–1500 °C. The mercury–cadmium–telluride (MCT) detection sensor was employed. The NETD was 25 mK at 25 °C. A lens with a focal distance of 25 mm (instantaneous field of view: 21.7° × 17.5°) was used.

For the evaluation of transient thermal responses, the transient profile of the temperature variations and temperature distributions on the black surface of the heat radiator was measured during heating for 3000 s at room temperature (25 °C). Similar to the numerical simulations, heating experiments were conducted by applying various bias voltages (10, 15, 20, and 22.5 V) at a sampling rate of 1 Hz.

We analyzed the NETD of the blackbody system based on the NETD of the infrared camera. In other words, the NETD of the blackbody system was evaluated in reverse using the NETD values of the infrared camera, which had already been precisely analyzed. The SiTF of the blackbody system was calculated as the slope value of a digital level in a thermal image. Digital levels were measured by applying bias voltages of 4–23 V in increments of 1 V, and linear fitting was used to calculate the slope value. The NETD consists of two components: the temporal NETD (NETD*_temp_*) and spatial NETD (NETD*_spat_*). The NETD is calculated from the experimentally obtained values of NETD*_temp_* and NETD*_spat_* using Equation (7):

(7) $$\mathrm{NETD}=\sqrt{{\left({\mathrm{NETD}}_{temp}\right)}^{2}+{\left({\mathrm{NETD}}_{spat}\right)}^{2}}.$$

We used the following image acquisition conditions to simultaneously obtain NETD*_temp_* and NETD*_spat_* in a single heating experiment. One hundred frames of thermal image data (640 × 480 pixels) with the interest region confined to 150 × 150 pixels (see Figure 2b) were acquired with a frame rate of 50 Hz at room temperature (22 °C). For NETD*_temp_*, one image data map consisting of 150 × 150 pixels was created by calculating the temporal standard deviation of each pixel for 100 frames. Then, NETD*_temp_* was calculated from the root mean square (RMS) information of this data map, according to Equation (8):

(8) $${\mathrm{NETD}}_{temp}=\frac{1}{\mathrm{SiTF}}\sqrt{\overset{n}{\underset{j=1}{\sum }}\frac{{\left(DV_{ij}-\bar{DV_{i}}\right)}^{2}}{n}}=\frac{\sigma_{TSV_{i}}}{\mathrm{SiTF}},$$

where *n* is the number of frames, *DV_ij_* is the digital level of the *i*-th pixel for the *j*-th frame, $\;\bar{DV_{i}}$ is the averaged digital level of the *i*-th pixel, and $\;\sigma_{TSV_{i}}$ is the temporal standard deviation of the digital level variation for each pixel.

For NETD*_spat_*, similar to NETD*_temp_*, one image data map (150 × 150 pixels) was reconstructed by calculating the mean of each pixel for 100 frames. Then, NETD*_spat_* value calculated using the RMS information of the map, according to Equation (9), is:

(9) $${\mathrm{NETD}}_{spat}=\frac{1}{\mathrm{SiTF}}\sqrt{\overset{n}{\underset{i=1}{\sum }}\frac{{\left(\bar{DV_{i}}-\bar{DV}\right)}^{2}}{n}}=\frac{\sigma_{SSV_{i}}}{\mathrm{SiTF}},$$

where $\;\bar{DV}$ is the averaged digital level for all pixels and $\;\sigma_{SSV_{i}}$ is the spatial standard deviation of the averaged digital level for each pixel.

Additionally, we evaluated the emissivity of the developed blackbody system. A portable emissometer (TSS-5X, Japan Sensor Corp., Tokyo, Japan) was used to repeatedly measure the emissivity of the black surface of the heat radiator at densely distributed measurement positions (100 mm intervals in 720 × 720 mm^2^) at room temperature (22 °C).

## 5. Results and Discussion

### 5.1. Transient Thermal Responses of Portable and Large-Area Blackbody System

**Figure 3:**
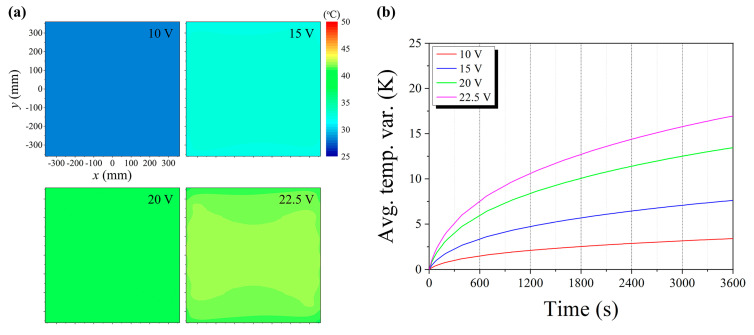
Numerical simulation results of the thermal responses of the portable and large-area blackbody system: (**a**) temperature distributions on the black surface; (**b**) transient profiles of the average temperature variations.

The crucial aspects of the thermal performance of a portable and large-area blackbody system are the uniformity of the temperature distribution on the heat radiator and the increase in radiant heat to a satisfactory level within a limited time. Therefore, we first analyzed the thermal responses of the designed blackbody system through numerical simulations, as described in Section 3. Figure 3a depicts the temperature distributions on the black surface of the heat radiator after heating for 3600 s by applying constant bias voltages (10, 15, 20, and 22.5 V). Almost uniform temperature distributions were observed on the black surface for all applied bias voltages. The differences between the maximum and minimum temperatures on the black surface were 0.14, 0.35, 0.67, and 0.95 °C for bias voltages of 10, 15, 20, and 22.5 V, respectively. Even though these temperature differences gradually increased with the applied bias voltage, the ratio of the temperature difference to the temperature variation averaged over the entire area was almost the same for all cases (approximately 0.09). The transient profiles of the average temperature variations until 3600 s are shown in Figure 3b. All curves analogously behaved in time. The temperature variations steeply increased for approximately 1800 s, and then, they gradually increased from 1800–3600 s. A temperature variation of more than 15 °C was obtained within 3600 s at a bias voltage of 22.5 V, which was our aim. Based on the above results, we confirmed that the proposed design was appropriate for ensuring the thermal performance of the blackbody system.

**Figure 4:**
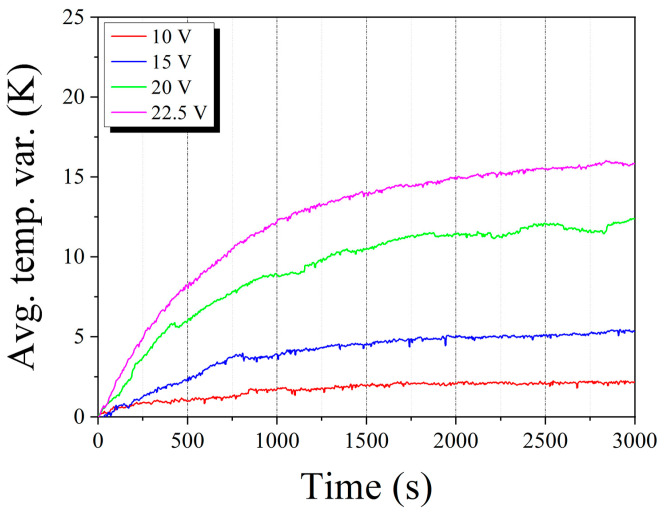
Measured transient profiles of average temperature variations.

**Figure 5:**
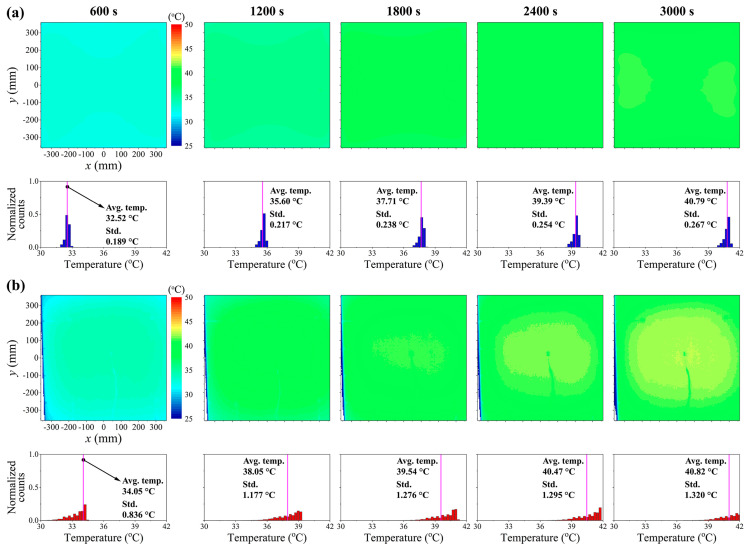
Transient temperature distributions and its histograms of the temperature values on the black surface of the portable and large-area blackbody system at a time interval of 600 s, for a bias voltage of 22.5 V: (**a**) numerical simulation; (**b**) experimental measurement. Note that because the total sampling numbers for the numerical simulation and experimental measurement differ from one another, the y-axis of the histograms indicated the normalized counts by calculating the number of the corresponding temperature value per sampling numbers.

Based on the effectiveness of the design determined via numerical simulations, we fabricated a prototype of the blackbody system as described in Section 2. Then, the thermal performance of the prototype system was evaluated using the thermal responses obtained via heating experiments. Figure 4 shows the experimentally measured transient profiles of the average temperature variations at different bias voltages. The profiles were similar to the numerical results at all bias voltages (refer Figure 3b), and a temperature increase of 15 °C was achieved at a bias voltage of 22.5 V. Figure 5 shows the transient temperature distributions and their histograms for the prototype of blackbody system at a time interval of 600 s, for a bias voltage of 22.5 V. As shown in the histograms of Figure 5a, the numerically calculated average temperatures at 600, 1200, 1800, 2400, and 3000 s were analyzed as 32.52, 35.60, 37.71, 39.39, and 40.79 °C, respectively. The measured average temperatures at 600, 1200, 1800, 2400, and 3000 s were estimated as 34.05, 38.05, 39.54, 40.47, and 40.82 °C, respectively (see Figure 5b). Based on the results of Figure 5, it was estimated that both the simulated and measured average temperatures increase were similar during an increasing time of 3000 s. In addition, the standard deviations of temperatures at each time show their uniform temperature distributions in both the simulation and measured results, even though the measured temperatures were more distributed than the simulation results as shown in the histograms of Figure 5a,b. Finally, both the simulated and measured average temperatures were almost the same at a heating time of 3000 s as 40.79 and 40.82 °C, respectively. Consequently, from these results, we validated the design and fabrication reliability of the blackbody prototype system as well as the targeted thermal performance of the blackbody system.

### 5.2. Signal Transfer Function (SiTF), Noise-Equivalent Temperature Difference (NETD), and Emissivity of Portable and Large-Area Blackbody System

**Figure 6:**
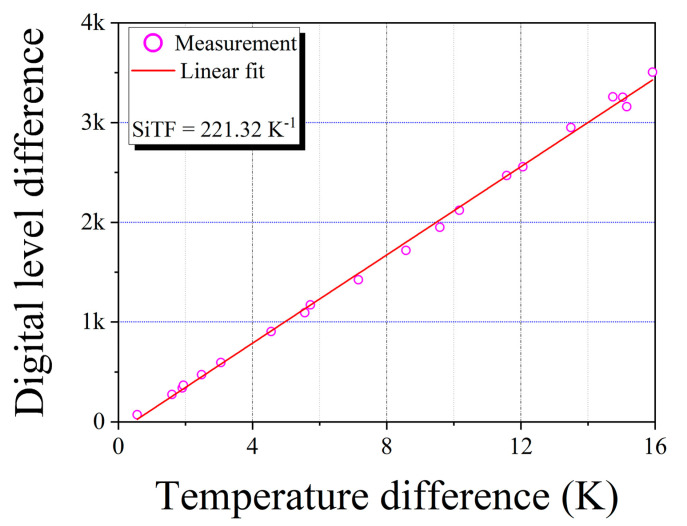
Signal transfer function (SiTF) curve of the portable and large-area blackbody system.

Figure 6 shows the digital level difference (*y*-axis) vs. the temperature difference (*x*-axis) between the measurement zone and the surrounding zone of the black surface of the heat radiator. The plot was acquired by increasing temperature. A straight line was fitted to the plot, the SiTF was determined from the slope of the line as 221.32 K^−1^.

**Figure 7:**
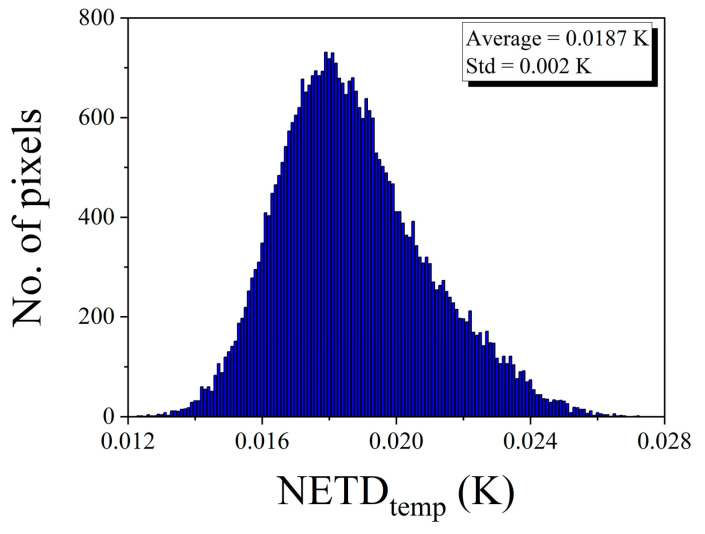
The histogram of temporal NETD of the portable and large-area blackbody system.

Figure 7 displays the histogram of the NETD*_temp_* values obtained in the region of interest (150 × 150 pixels) for all stored frames. NETD*_temp_* showed a Gaussian distribution over a range of 12–27 mK, and the average NETD*_temp_* was 18.7 mK. Additionally, the average NETD*_spat_* was calculated as 31.62 mK.

**Figure 8:**
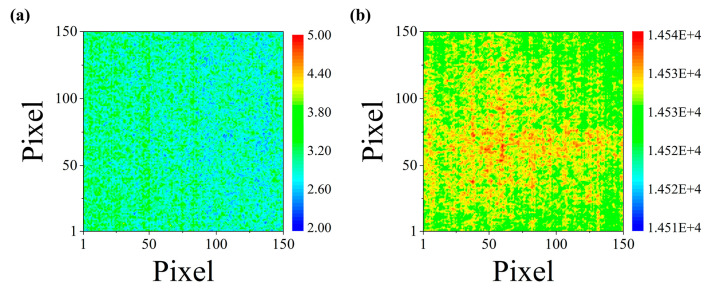
Reconstructed image data maps of (**a**) standard deviation and (**b**) average of each pixel for 100 frames of thermal image data.

Figure 8a,b show the image data maps (150 × 150 pixels) for NETD*_temp_* and NETD*_spat_*, respectively; the maps were obtained based on the experimental procedures described in Section 4. These figures represent the values of the standard deviation and average of each pixel for 100 frames of the thermal image. These image data maps were utilized to calculate the NETD using Equation (7). It was verified that the NETD of the infrared camera of the developed blackbody system was 36.74 mK. Moreover, the mean and standard deviation of the emissivity evaluated at all measurement positions on the black surface of the heat radiator were 0.987 and 0.0118, respectively.

## 6. Conclusions

A portable and large-area blackbody system was designed and fabricated, and its thermal performance was evaluated. The system was lightweight (5 kg) so that it could be portable. This differentiates the developed system from existing small-sized blackbody systems that are commonly used in laboratory environments. The design was validated based on transient coupled electro-thermal simulation results, which showed fairly good thermal characteristics such as the uniformity of the temperature distribution on the surface of the heat radiator and increase in radiant heat to a satisfactory level within a limited time. Then, a prototype of the blackbody system was fabricated and its operational performance (thermal responses, SiTF, NETD, and emissivity) was verified using an infrared thermal imaging system and a portable emissometer. Based on the thermal responses of the portable and large-area blackbody system obtained in this study, we believe that this system can be expected to serve as a fairly stable reference source for the calibration of aerial infrared cameras or infrared sensors embedded in UAVs over their working temperature ranges.

## Figures and Tables

**Table 1:** Electrical and thermal properties used for the finite element analysis.

	Density, *ρ* (kg/m^3^)	Specific Heat Capacity, *C_p_* (J/kg·K)	Thermal Conductivity, *k* (W/m·K)	Electric Conductivity, *σ* (S/m)	Dielectric Constant
Black aluminum plate [30,31,32,33,34]	Temperature-dependent *ρ*; 2736.893 − (6.011681 × 10^−3^ × *T*) − (7.012444 × 10^−4^ × *T*^2^) + (1.3582 × 10^−6^ × *T*^3^) − (1.367828 × 10^−9^ × *T*^4^) + (5.177991 × 10^−13^ × *T*^5^) Temperature-dependent *C_p_*; 595.6585 + (1.513029 × *T*) − (2.070065 × 10^−3^ × *T*^2^) + (1.303608 × 10^−6^ × *T*^3^) Temperature-dependent *k*; 39.646 + (1.684 × *T*) − (5.4134 × 10^−3^ × *T*^2^) + (8.4313 × 10^−6^ × *T*^3^) − (6.537 × 10^−9^ × *T*^4^) + (2.002 × 10^−12^ × *T*^5^)				
Polyethylene [35]	930	1900	0.38	-	-
Acrylic frame	1190	1470	0.18	-	-
Carbon-impregnated cotton yarn (CICY)	50	0.5	3000	1550	1
Thermoplastic polyurethane [36]	1250	1674	Temperature-dependent *k*; 0.197 + (3.349 × 10^−4^ × *T*)	-	-
Copper wire woven with the CICY	100	100	30,000	2300	1
